# A rare case of Guillain-Barrè syndrome with unilateral facial palsy and asymmetrical ascending weakness: a case report

**DOI:** 10.1097/MS9.0000000000000868

**Published:** 2023-06-07

**Authors:** Suman Maharjan, Birat Bhattarai, Sanjog Basnet, Suvekchya Pandey, Sarita Basnet, Pawan Shrestha, Kriti Thapa

**Affiliations:** a College of Medicine, Nepalese Army Institute of Health Sciences (NAIHS), Sanobharyang; b Department of Medicine, Shree Birendra Hospital, Chhauni, Kathmandu, Nepal

**Keywords:** asymmetrical ascending weakness, case report, facial palsy, guillain-barrè syndrome

## Abstract

Guillain-Barrè syndrome is a life threatening postinfectious disease causing acute paralytic neuropathy that rarely presents with asymmetrical limb weakness (1%) and unilateral facial nerve palsy (4.9%).

**Case Presentation:**

A 39-year-old male presented with pain and weakness in the right lower limb with right sided facial weakness. The cranial nerve examination revealed lower motor neuron type right facial palsy (Bell ’s palsy). On rest neurological examination, he had decreased power in the right lower limb with an absent knee and ankle reflex on presentation. Later, the weakness was symmetrical in both lower limbs.

**Clinical Discussion:**

Cerebrospinal fluid analysis showed albuminocytologic dissociation with no cells and an elevated protein of 203.2 mg/dl. The nerve conduction study was abnormal in bilateral lower limbs suggesting severe demyelinating motor neuropathy. Intravenous Immunoglobulin was started with the dose of 25 gm (0.4 mg/kg) once daily for 5 days, that is, five doses. The patient started showing signs of recovery with the initial dose of immunoglobulin.

**Conclusion:**

The course of the disease usually recovers spontaneously and completely; however, plasma exchange and immunomodulatory therapy have shown improvement in patient with rapidly deteriorating symptoms.

## Introduction

Highlights

- Guillain-Barrè syndrome (GBS) is a life threatening postinfectious disease and one of the most common cause of acute paralytic neuropathy (0.4–2 per 100 000 population) that presents with a triad of progressive motor weakness, areflexia, and albuminocytologic dissociation.
- GBS also frequently involves the cranial nerve (45–75%) especially facial nerve palsy bilaterally; however, unilateral palsy is uncommon in adults (only in 4.9%).
- Asymmetrical limb weakness presentation in cases of GBS remains as low as 1%.
- The diagnosis of GBS is based upon clinical presentation, neurological, electrophysical, and cerebrospinal fluid examinations. Suspected GBS undergo lumber puncture, which show cyto-albuminological dissociation with increased protein count and normal cell count.
- The course usually shows spontaneous and complete recovery; however, immunomodulatory therapy should be started if the patient rapidly deteriorates.

Guillain-Barrè syndrome (GBS) is a life threatening postinfectious disease and one of the most common cause of acute paralytic neuropathy (0.4–2 per 100 000 population) that presents with a triad of progressive motor weakness, areflexia, and albuminocytologic dissociation^1–4^. GBS mostly presents acutely and progess rapidly with symmetrical sensory symptoms that start distally^5^. Asymmetrical limb weakness presentation in cases of GBS remains as low as 1%^6^. GBS also frequently involves the cranial nerve (45–75%) especially facial nerve palsy bilaterally; however, unilateral palsy is uncommon in adults (only in 4.9%)^1,7–9^.

We report a case of a 39-year-old man who initially presented with asymmetrical ascending weakness starting from the muscles of the right lower limb, which later became bilateral in addition to bilateral upper limb weakness including asymmetrical right facial nerve paralysis.

## Method

We reported this case in line with the updated consensus-based Surgical CAse REport (SCARE) 2020 criteria^10^.

## Case presentation

A 39-year-old male with no known co-morbidities presented to the Emergency Department of our hospital with complaints of pain and weakness in the right lower limb. The pain was gradual in onset, pricking in character, radiating to the lower back, aggravated on activities/ exertion and also in the sitting position, which relieved on rest and lying down. The patient also presented with facial weakness in the right side. He also complained of a history of fever 15 days before the presentation in our hospital. According to him, he was treated as enteric fever; however, proper documentation of the treatment was not available. His family history, recent travel history, and allergic history were non remarkable. He does not consume alcohol, has a normal bowel habit, and is a reformed smoker.

On admission, he was alert, well oriented to time, place, and person with a Glasgow Coma Scale of 15. His vital parameters were stable on arrival. Initial baseline blood works were within the normal range including random blood sugar. His higher mental functions were appropriate. The cranial nerve examination revealed lower motor neuron type right facial palsy (Bell’s Palsy) showing right facial droop with loss of the right nasolabial fold, inability to close the right eye, inability to raise right the eyebrow and deviation of the angle of the mouth towards the left side. On rest of the neurological examination, he had decreased touch sensation from L1 to L5 dermatome of the right side with normal vibration and normal joint position sensation (proprioception). He had decreased power (3/5) of the right lower limb muscles supplied by L1–L3. Initially, there was asymmetrical muscle weakness in his lower limb, which progressed to symmetrical weakness of 3/5 power in both lower limbs and 4/5 in both upper limbs. Ankle and knee joint reflex was absent on the right however normal bilateral biceps and triceps reflex with preserved gag reflex, perianal sensation, anal wink, and anal tone. There was a bilateral upward response on the plantar reflex. There were no signs of meningeal irritation and absent cerebellar signs. Patient later complained of dysphagia. Other systemic examination including respiratory, gastrointestinal, and cardiovascular were normal.

Cerebrospinal fluid (CSF) analysis showed albuminocytologic dissociation with no cells and an elevated protein of 203.2 mg/dl with no pathogenic growth on CSF, blood, and urine culture. Nerve conduction study was abnormal in bilateral lower limbs suggesting severe demyelinating motor neuropathy. MRI of the head and spine showed nonspecific changes in brain and only mild degenerative disease in lumbar spine.

Intravenous immunoglobulin (IVIG) was started with the dose of 25 gm (0.4 mg/kg) once daily for 5 days, that is, five doses.The patient started showing signs of recovery with the initial dose of immunoglobulin. The patient was monitored for 2 more weeks in the hospital and during the observation the patient’s lower limb weakness, upper limb weakness, and facial palsy drastically improved. The patient was then discharged (2 weeks after the admisistration of IVIG). At the time of discharge the patient had a power of 4/5 on both lower limbs with 5/5 power of the upper limbs and the facial palsy completely disappeared. The pain and weakness of the lower extremities partially resolved so was planned for schedule follow-up in the outpatient department. A follow-up was done 3 months after the discharge and the patient still had slight pain and weakness on lower limb; however, could carry out his normal day to day activities.

## Discussion

GBS affects the myelin sheath and Schwann cells called acute inflammatory demyelinating polyneuropathy or can either be an axonal variant called acute motor axonal neuropathy or acute motor – sensory axonal neuropathy^2,11^. The exact cause of GBS is unknown; however, 50–70% appear 1–2 weeks after a respiratory or gastrointestinal infection (typically occurs after infection from campylobacter jejuni, mycoplasma pneumonia, influenza virus, epstein virus, or cytomegalovirus), or an autoimmune response against peripheral nerves and their spinal roots^2,12^.

Symptoms in GBS can develop within days (acutely) or even within weeks (subacutely) and most reach peak within 2–4 weeks^12^. Typically, GBS presents with rapidly progressive bilateral weakness that starts in the distal lower extremities^13^; however, our case initially presented with asymmetrical ascending lower limb weakness, which occurs in about 1%^6^. The nerve involvement is great in distal nerves and at sites of entrapment due to relative blood nerve barrier deficient allowing antibodies access^1,14^.

Cranial neuropathy is common in GBS with bulbar palsy being the most common presentation (49.2%) with symptoms of dysphagia. Bulbar palsy is an indicator of respiratory paralysis. 46% of patients present with facial palsy bilateral; however, unilateral involvement is rare (4.9%). The uncommon unilateral involvement of the facial nerve is secondary to the development of antibodies directly resulting in either demyelination or axonal degeneration^15^.

The diagnosis of GBS is based upon clinical presentation, neurological, electrophysical, and CSF examinations^3^. Suspected GBS undergo lumber puncture, which show cyto-albuminological dissociation with increased protein count and normal cell count. The protein count in GBS increases by the end of the second week^2^. 57% of Bell’s palsy have pathological enhancement of the facial nerve in head enhanced MRI, which is absent in GBS that helps in distinguishing^7^. There were no such specific signs present in our case. Electrodiagnostic studies help supporting the diagnosis and help differentiate between axonal and demyelinationg subtypes of GBS^3,13^. However, the measurements can remain within the normal range when performed earlier in the disease course (within 1 weeks)^3^.

The course usually shows spontaneous and complete recovery; however, immunomodulatory therapy should be started if the patient develops progressive weakness rapidly or if other symptoms like autonomic dysfunction, bulbar failure, respiratory insufficiency, or if the patient is unable to walk^3,12^. IVIg, when started within 2 weeks of the onset of weakness shows a significant effect. 0.4 g/kg body weight daily for 5 days are effective dose for the treatment of GBS^3^. Plasma exchange is equally effective; however, IVIg is easier to administer and more widely available^3,12^. The distal limb muscles recover slower than that of the muscles innervated by the cranial nerve^8^. 80% of the patients have full recovery within 12 months^4^. Although favorable prognosis, mortality ranges from 2 to 12% with 15% having persistent disability and 20–30% requiring mechanical ventilation^4,12,13^.

## Conclusion

In the study, we reported a rare case of GBS with unilateral facial palsy and asymmetrical ascending paralysis at the same time. Although an unusual presentation, the physician should possess a high degree of suspicion along with a proper history, clinical, neurological, electrophysical, cerebrospinal fluid examination, and imaging modalities. The course of the disease usually recovers spontaneously and completely; however, in few cases plasma exchange and immunomodulatory therapy have shown improvement especially in patient with rapidly deteriorating symptoms.

## Ethical approval

None.

## Consent

Written informed consent was obtained from the patient for publication of this case report. A copy is available for review by the Editor-in-chief of this journal on request.

**Written consent from the patient:** Written informed consent was obtained from the patient for publication of this case report and accompanying images. A copy of the written consent is available for review by the Editor-in-Chief of this journal on request.

## Source of funding

None.

## Author contribution

S.M., B.B., S.B., S.P., S.B., P.S., and K.T. were involved in the management of the patient and involved in writing, editing, and review of the manuscript. All authors read and approved the final manuscript.

## Conflicts of interest disclosure

None.

## Research registration unique identifying number (UIN)

1. Name of the registry: NA.
2. Unique Identifying number or registration ID: NA.
3. Hyperlink to your specific registration (must be publicly accessible and will be checked): NA.

## Guarantor

Suman Maharjan.

## Data availability statement

Not applicable.

## Provenance and peer review

Not commissioned, externally peer reviewed.
